# TAS2R38 bitter taste genotype is associated with complementary feeding behavior in infants

**DOI:** 10.1186/s12263-019-0640-z

**Published:** 2019-05-03

**Authors:** Gabriele Cont, Giulia Paviotti, Marcella Montico, Paola Paganin, Martina Guerra, Antonella Trappan, Sergio Demarini, Paolo Gasparini, Antonietta Robino

**Affiliations:** 1 Institute for Maternal and Child Health–IRCCS “Burlo Garofolo”, Via dell’Istria 65/1, 34137 Trieste, Italy; 2 Department of Medical Sciences, University of Trieste, Strada di Fiume, 447, 34149 Trieste, Italy

**Keywords:** TAS2R38, Infants, Complementary food

## Abstract

**Background:**

Genetically mediated sensitivity to bitter taste has been associated with food preferences and eating behavior in adults and children. The aim of this study was to assess the association between TAS2R38 bitter taste genotype and the first complementary food acceptance in infants.

Parents of healthy, breastfed, term-born infants were instructed, at discharge from the nursery, to feed their baby with a first complementary meal of 150 mL at 4 to 6 months of age. They recorded the day when the child ate the whole meal in a questionnaire. Additional data included food composition, breastfeeding duration, feeding practices, and growth at 6 months. Infants’ TAS2R38 genotypes were determined at birth, and infants were classified as “bitter-insensitive” (genotype AVI/AVI) and “bitter-sensitive” (genotypes AVI/PAV or PAV/PAV).

**Results:**

One hundred seventy-six infants and their mothers were enrolled; completed data were available for 131/176 (74.4%) infants (gestational age 39.3 ± 1.1 weeks, birth weight 3390 ± 430 g). Bitter-insensitive were 45/131 (34.3%), and bitter-sensitive were 86/131 (65.6%). Thirty-one percent of bitter-insensitive infants consumed the whole complementary meal at first attempt, versus 13% of bitter-sensitive ones (*p* = 0.006). This difference was significant independently of confounding variables such as sex, breastfeeding, or foods used in the meal. Growth at 6 months did not differ between the two groups.

**Conclusions:**

Differences in *TAS2R38* bitter taste gene were associated with acceptance of the first complementary food in infants, suggesting a possible involvement in eating behavior at weaning.

## Background

Complementary food introduction marks the beginning of the transition between an exclusively milk-based diet and family food consumption. Acceptance of the first complementary food varies among children and is influenced by several factors [1], including both environmental and genetic ones [2]. Taste is one of the most important factors in determining food preference in both children and adults. The sensory experience of foods certainly plays a role in their initial acceptance, and some tastes are more acceptable than others [3]. For instance, an innate preference for sweet taste and an innate rejection for bitter taste have been reported, which can in turn influence likes and dislikes of foods [4–6]. Studies on infants at weaning time (5–7 months old) reported that vegetables with salt are more accepted than vegetables without salt or that the acceptance of sweet, umami, or sour foods is linked to the preference for solutions of the same taste [7–9]. Concerning bitter taste perception, variations in the gene coding for bitter receptors were associated to interindividual differences in taste perception and evidences showed that these differences can affect eating behavior especially in children [10]. Among the 25 bitter taste receptors (TAS2R), the most widely studied has been TAS2R38, which is responsible for differences in taste perception of two bitter compounds, phenylthiocarbamide (PTC), and 6-n-propylthiouracil (PROP) [11, 12]. Individuals who perceive these compounds moderately or intensely bitter (about 75%) are considered “medium tasters” or “super tasters” while those who perceive these compounds as weak or tasteless are considered “non-tasters” (about 25%) [13].

Three polymorphisms in the TAS2R38 gene result in three amino acid substitutions defining two major haplotypes, PAV and AVI. Subjects carrying at least one copy of the dominant PAV are mainly medium taster or super taster, while AVI homozygote individuals are non-tasters [11, 12, 14]. However, evidences also suggested that polymorphisms in the *TAS2R38* gene partly explain variance in PTC/PROP perception and a role by other genetic and non-genetic factors has been suggested [15, 16].

Studies conducted in both adults and children reported a relationship between PTC/PROP taste perception, mediated by the TAS2R38 gene, and eating behavior [17–22]. Basically, non-taster individuals result less sensitive to a wider range of taste stimuli and show a greater acceptance of different foods, such as cruciferous raw vegetables, spicy foods, or alcoholic beverages [23–26]. Moreover, TAS2R38 genotype has been associated with preference and intake for sucrose and sweet foods. Specifically, sweet food preference and intake resulted higher in children homozygous for the bitter-sensitive allele or heterozygous compared to children homozygous for the bitter-insensitive allele [10, 27]. Interestingly, a recent study showed that variants in TAS2R38 gene are associated to pick eating behavior with children carrying at least one copy of the bitter-sensitive allele having limited dietary variety compared to the homozygous for bitter insensitive allele [28].

Despite the already existing research studies on genetic differences in taste perception and food behavior, no data is available on the possible role of TAS2R38 genotype on the acceptance of first complementary food in infants. Therefore, the aim of this study was to determine if TAS2R38 genotype affects complementary feeding behavior in infants, hypothesizing that less time is needed to accept the first complementary foods in bitter-insensitive infants if compared to bitter-sensitive ones.

## Methods

### Subjects

A prospective cohort study was carried out at the Institute for Maternal and Child Health–IRCCS “Burlo Garofolo,” Trieste. One hundred seventy-six mothers and their healthy infants of Italian parents, born at ≥ 37 gestational weeks, with a birth weight ≥ 2500 g, were recruited from November 2013 to July 2015 in the nursery. The main exclusion criteria were the need for resuscitation or admission to intensive care unit, congenital malformations, exclusive formula feeding, non-Italian parents, residency outside the Trieste area, and lack of parental consent.

After informed consent was obtained from mothers, two samples of buccal mucosa were retrieved for genetic analysis, by gentle brushing the internal surface of the cheek with a swab (Isohelix, Cell Projects, Kent, UK) for 2 min on each side.

### Feeding information

After enrollment, mothers received a written copy of a questionnaire containing the following items: name, surname, and family address; name of pediatrician; anthropometric measures (weight, length, and head circumference) at birth and at 6 months of age (as measured by pediatrician at health check); type of feeding at hospital discharge (exclusive or partial breastfeeding); duration of breastfeeding; day of first introduction to complementary food; day when the baby ate the whole volume offered (150 mL); parental rating (0 to 10 score) of the baby’s liking of food; complementary food composition including the frequency of each food added (always, sometimes, never); and who fed the baby. The Infant Feeding Questionnaire (IFQ) was also administered to assess maternal feeding practices and beliefs. The rating scales for questions on feeding practices were anchored by the terms “never” and “always.” The rating scales for questions on feeding beliefs were anchored by the terms “disagree a lot” and “agree a lot” [29].

Mothers were instructed how to complete the questionnaire and on the method of feeding at the introduction of the complementary foods. Specifically, mothers were asked to give their baby a fixed volume of food (150 mL), measured with a graduated bottle. The composition of the first complementary food was based on a standard traditional local recipe (i.e., a semi-liquid vegetable soup made with mashed carrots, potatoes, and zucchini, plus parmesan and oil). Moreover, additional foods could be added: cereal powder (wheat, rice, oat, corn, tapioca), meat, fish, legumes, and other vegetables (such as spinach, chard, brassicaceae, pumpkins, tomato). The frequency of each added food was reported by mothers in the questionnaire. Whenever used, the frequency of adding salt was also recorded. Small quantities of mashed or homogenized fruit, given in between milk feeds, were not considered. The volume of 150 mL of food was chosen to approximately match a typical full meal of a 6-month old infant [30].

Four months after the delivery, the introduction of complementary foods might start [31]. Mothers were contacted and reminded to offer 150 mL of complementary food and to fill in the questionnaire, with particular regard to the date in which the baby ate the whole volume of food.

When infants reached the objective to eat the whole volume of food, phone calls and home visits were organized in order to collect the questionnaires with the data.

### Genetic analysis

DNA was extracted using Isohelix extraction protocol-DNA isolation kit (Cell Projects, Kent, UK). Genotypes of three SNPs located within the TAS2R38 gene (rs1726866, rs713598, and rs10246939) were defined using the TaqMan probe-based assays (Applied Biosystems, Foster City, CA, USA). Based on the results, all participants were then classified into heterozygous PAV/AVI, homozygous PAV/PAV, and homozygous AVI/AVI. For statistical analysis, AVI/AVI infants were considered as “bitter-insensitive,” while PAV/PAV and PAV/AVI infants as “bitter-sensitive.”

### Statistical analysis

Variables were reported as mean (± standard deviation) or median (interquartile range) if normally or non-normally distributed, respectively. Normally distributed continuous variables were compared with *t* test for independent samples, while non-normally distributed variables were compared with the Mann-Whitney test. Proportions were compared by the chi-square test. To assess the independent association between the proportion of infants who consumed all 150 mL of food on the first day and bitter taste genotype, a backward stepwise logistic regression analysis was conducted; the following variables were included: ongoing breastfeeding, gestational age at birth, infants feeding practices scores, sex, days of life at first complementary food introduction, complementary foods used in the meal, and primipara/multipara mother. Only variables associated with a *p* < 0.1 were retained in the model. Statistical significance was assumed at *p* < 0.05. Statistical analyses were performed using R.

## Results

One hundred seventy-six infants and their mothers were enrolled at birth, but complete data were available only for 131 of them (74.4%). They were born at 39.3 ± 1.1 weeks of gestational age, with a birth weight of 3390 ± 430 g, length 50 ± 2 cm, and cranial circumference 34.4 ± 1.3 cm. Males were 71/131 (54.2%), and first-born infants were 96/131 (73.3%). At discharge, 22 infants (16.8%) were partially breastfed while 109 (83.2%) exclusively. At the time of the first complementary food introduction, 90 out of 131 (68.7%) were still breastfed, in agreement with data already published [32]. Bitter-insensitive infants (genotype AVI/AVI) were 45/131 (34.3%), while 86/131 (65.6%) were bitter-sensitive, divided as follows: 28.2% PAV/PAV (37/131) and 37.4% PAV/AVI (49/131). Characteristics at birth and at the time of first complementary food introduction did not differ between bitter-insensitive and bitter-sensitive infants (Table 1). Complementary food was first introduced at 167 ± 20 days of life in bitter-insensitive and 167 ± 24 days in bitter-sensitive (*p* = 0.9). Seventy-two percent of bitter-sensitive infants and 62% of bitter-insensitive infants were still breastfed (*p* = 0.3).

**Table 1:** Sample characteristics at birth and at the time of first complementary food introduction

	Bitter-sensitive infants (*n* = 86)	Bitter-insensitive infants (*n* = 45)
At birth		
Gestational age (weeks)	39.3 ± 1.1	39.4 ± 1.4
First-born	61/86 (70.9%)	35/45 (77.8%)
Males	43/86 (50%)	28/45 (62.2%)
Weight (g)	3370 ± 440	3420 ± 400
Length (cm)	50 ± 2	50 ± 2
Cranial circumference (cm)	34.4 ± 1.2	34.7 ± 1.3
Exclusive breastfeeding	76/86 (88.4%)	33/45 (73.3%)
At the time of first complementary food introduction		
Days of life at first complementary food introduction	167 ± 24	167 ± 20
Weight at 6 months (g)	7875 ± 899	8045 ± 1157
Length at 6 months (cm)	68.3 ± 3.4	68.4 ± 3.7
Cranial circumference at 6 months (cm)	43.1 ± 1.7	43.5 ± 2.2
Ongoing breastfeeding	62/86 (72%)	28/45 (62%)

Significance: *p* > 0.5 for all comparisons

Data are reported as mean ± standard deviation or number of cases/total (percentage)

Bitter-sensitive infants refers to AVI/PAV or PAV/PAV genotype, while bitter-insensitive to AVI/AVI genotype

Frequency of food intakes as part of the first complementary food mix did not differ between bitter-sensitive and bitter-insensitive infants as reported in Table 2.

**Table 2:** Frequency of food intake as part of complementary food mix by bitter-sensitive and bitter-insensitive infants

Food	Bitter-sensitive (*n* = 86)			Bitter-insensitive (*n* = 45)		
	Always (%)	Sometimes (%)	Never (%)	Always (%)	Sometimes (%)	Never (%)
Carrots	77.7	21.1	1.2	82.2	17.8	0
Zucchini	67.0	28.2	4.8	62.3	33.3	4.4
Pumpkins	4.7	51.7	43.6	13.3	33.3	53.4
Brassicaceae	2.4	44.7	52.9	8.9	35.6	55.5
Spinach/chard	18.9	60	21.1	22.3	53.3	24.4
Potatoes	61.2	34.1	4.7	64.4	33.3	2.3
Legumes	3.5	58.9	37.6	4.4	46.7	48.9
Tomato	0	4.7	95.3	0	6.7	93.3
Meat	27.9	54.7	17.4	31.1	53.3	15.6
Fish	9.3	54.7	36.0	8.9	51.1	40.0
Cereal	23.5	41.6	34.9	20	43.3	36.7
Oil	87.2	12.8	0	86.7	13.3	0
Parmesan	66.3	32.6	1.1	75.6	22.2	2.2
Salt	11.6	23.3	65.1	6.7	28.9	64.4
Sugar	0	9.3	90.7	0	6.7	93.3

Brassicaceae includes cabbage, cauliflower, broccoli, and Brussels sprouts. Cereal includes wheat, rice, oat, or mixed cereals (including also corn and tapioca)

*p* > 0.05 in all comparisons of percentages of bitter-sensitive versus bitter-insensitive infants

The whole volume (150 mL) of complementary food was consumed at the first attempt by 14/45 (31%) bitter-insensitive infants and by 11/86 (13%) bitter-sensitive ones (*p* = 0.006). This difference remained significant after correcting for the following variables: food composition, food liking, maternal feeding practices and belief, ongoing breastfeeding at the introduction of complementary food, infant’s age at complementary food introduction, gestational age at birth, and sex (Table 3: stepwise logistic regression). Bitter-insensitive infants ate the whole volume of food (150 mL) after 6 (0–22) days, whereas bitter-sensitive ones ate it after 10 (2–27) days (*p* = 0.05). The score assigned by parents to the baby’s liking of food was 7.7 ± 1.6 in bitter-sensitive and 7.8 ± 2.0 in bitter-insensitive (*p* = 0.8).

**Table 3:** Independent variables associated with whole first meal consumption at the first attempt

Independent variables	Adjusted OR (95% CI)	*p* value
Bitter-insensitive vs bitter-sensitive	4.29 (1.55–12.6)	0.006
Days of life at first complementary food introduction	0.98 (0.97–1.00)	0.02
Meat	2.54 (1.17–5.90)	0.02
Pumpkin	0.41 (0.16–0.94)	0.04
Salt	0.27 (0.07–0.73)	0.02

Dependent variable: consumption of whole volume (150 mL) of complementary food at first attempt (yes vs no)

Excluded variables were (*p* > 0.05) ongoing breastfeeding, gestational age, maternal feeding practices and beliefs, sex, primipara/multipara mother, liking of food, and other food components (oil, parmesan, fish, sugar, legumes, zucchini, carrots, potatoes, spinach/chard, tomato, cereal, brassicaceae)

Complementary food components are reported in order of descending frequency (always-sometimes-never). *OR* odds ratio

Meat in complementary food independently increased the odds of consuming the whole meal at the first attempt (adjusted OR 2.54, *p* = 0.02). Conversely, salt decreased such odds (adjusted OR 0.27, *p* = 0.02). Moreover, maternal feeding practices on child eating behaviors and maternal belief assessed by IFQ did not affect the acceptance of meal at the first attempt (*p* = 0.09 and *p* = 0.60).

At 6 months, anthropometric measures of bitter-sensitive infants did not differ to those of bitter-insensitive ones: weight 7875 ± 899 g versus 8050 ± 1160 g (*p* = 0.4), length 68.3 ± 3.4 cm versus 68.4 ± 3.7 cm (*p* = 0.9), and cranial circumference 43.1 ± 1.7 cm versus 43.5 ± 2.2 cm (*p* = 0.3).

## Discussion

This study indicates that infants insensitive to bitter taste (defined by the TAS2R38 genotype AVI/AVI) were more likely to consume the whole first complementary food meal at first attempt, compared to sensitive ones (either AVI/PAV or PAV/PAV genotypes). Moreover, bitter-insensitive infants consumed the whole volume of the first complementary food in fewer days than bitter-sensitive ones.

To our knowledge, this is the first study to show an association between TAS2R38 genotype and acceptance of complementary food in infants.

Previous research already suggested a link between differences in foods preferences and intake (especially for sugar and sweet foods) and genetic sensitivity to bitter taste [10]. Interestingly, our finding agrees with recent work showing that variants in the TAS2R38 gene are associated to pick eating behavior in 2–5-year-old children [28]. Authors showed that homozygous bitter-sensitive children have limited dietary variety and tend to be picky eaters compared to homozygous bitter-insensitive children. In line with this work, we found that in homozygous bitter-insensitive infants, less time is needed to accept the first complementary foods.

In the present study, TAS2R38 haplotype distribution was similar to that reported for other Caucasian populations [33], making our cohort well representative of the whole population.

Bitter-sensitive and bitter-insensitive infants were similar with respect to baseline features and to the timing of first complementary food introduction. Infants were all breastfed, at least partially. Exclusive formula-fed infants were excluded because, in comparison to them, breastfed infants are more willing to try and accept new foods [34] as they experience flavors derived from the maternal diet in breast milk [35].

As the attitude adopted by the person who feeds the infant may influence food consumption [35], the study protocol included an IFQ designed for mothers (maternal feeding practices and maternal beliefs). In our study population, all infants were fed by their own mothers and the IFQ items were not significantly associated with first food consumption.

Moreover, no differences emerged in specific foods as part of the complementary food mix between bitter-sensitive and bitter-insensitive infants. Interestingly, the presence of meat in complementary food independently increased the odds of consuming the whole meal at the first attempt, whereas adding salt decreased such odds.

An association between individual differences in PROP/PTC bitter taste perception and body mass index has been reported in both adults and children [36–38]. However, we did not find any significant correlation between TAS2R38 bitter taste genotype and infants’ growth at 6 months of age. This result was largely expected, as it is unlikely that a few days difference in the consumption of 150 mL of complementary food between bitter-sensitive and bitter-insensitive infants may influence short-term growth. To assess the impact of bitter taste genetics on growth, anthropometric measurements should be evaluated at longer intervals from complementary food introduction.

Past works have mainly focused on the role of repeated exposure, breastfeeding, or weaning practices [1, 39–41]. Our study showed for the first time that polymorphisms associated with PROP bitter taste perception are among the factors that may influence food acceptance at the beginning of weaning. Since complementary feeding represents a very important step in the development of food behavior, it is likely to affect the infant’s nutritional status and health later in life [42–45]. Thus, the findings of this study can assist in identifying infants or groups of infants who are less likely to comply with the acceptance of complementary foods, and as consequence help mothers with additional guidance for infant feeding.

Despite the interesting findings we identified, we should acknowledge that the study has some limitations: (1) The complementary food composition was based on a common local recipe, and thus, results might be relevant for people living in North-Eastern Italy. As matter of facts, (a) in our geographic area, first complementary foods are prepared in a rather uniform way; (b) only Italian families were enrolled. (2) The volume of 150 mL of food was chosen arbitrarily. Although this volume of food was not validated previously in research settings, a 150 ml volume approximately matches a full meal of a 6-month old infant [30]. (3) The study was based on self-reporting by parents, and thus, reporting errors cannot be excluded. However, there is no reason to believe that reporting errors differentially affected the two groups. (4) We only studied the impact of TAS2R38 bitter taste genotype on complementary food acceptance while genes coding for other taste receptors may also play a role.

For all the abovementioned reasons, additional studies on larger populations are required to confirm our findings and to evaluate the impact of different volumes of complementary food or of different types of food.

## Conclusions

The study results agree with our initial hypothesis and are also in line with already reported differences in food behavior between bitter-sensitive and bitter-insensitive adults and children [28, 46, 47].

This work, through a better understanding of the genetics of taste on eating behavior, represents a potential starting point for further investigation and aims at developing positive eating habits at weaning.

## Abbreviations

IFQ: Infant Feeding Questionnaire
PROP: 6-n-Propylthiouracil
PTC: Phenylthiocarbamide
SNPs: Single nucleotide polymorphisms
